# Concepts and Issues in COA Research

**Published:** 1997

**Authors:** Michael Windle

**Affiliations:** Michael Windle, Ph.D., is professor of psychology and director of the Doctoral Studies Program in Developmental Psychology at the University of Alabama at Birmingham

**Keywords:** AODD (alcohol and other drug dependence), children of alcoholics, epidemiology, study design, sample selection, data collection, identification and screening for AOD use, family AODU (alcohol and other drug use) history, risk factors, causes of AODU, scientific model, literature review

## Abstract

Estimates of the number of children of alcoholics (COA’s) and the prevalence of alcohol use disorders among them can vary widely from study to study depending on research design features such as sample selection, data collection strategies, and assessment methods. Although investigators agree that COA’s are at higher risk for developing alcohol use disorders than children of nonalcoholics, problems with alcohol are not an inevitable consequence of COA status. Recent research has identified numerous biological, psychological, and social factors associated with a family history of alcoholism that may play a role in determining whether COA’s will develop an alcohol use disorder. The conceptual model presented in this article gives a general overview of how such risk factors can interact with life stressors to influence alcohol-related behavior in COA’s. (Subsequent articles in this issue explore some of the specific factors identified in the model in greater depth.)

For years common wisdom has fostered the notion that alcoholism runs in families. To a large extent, recent scientific studies have substantiated this concept, although with two important provisos (for reviews, see Sher 1991; West and Prinz 1987; Windle and Searles 1990). First, not all children of alcoholics (COA’s) develop alcohol use disorders^1^ or other forms of psychopathology, such as depressive disorders; hence, the manifestation of an alcohol use disorder or other psychopathology is not inevitably associated with COA status. Second, both COA’s and children of nonalcoholics (non-COA’s) substantially overlap in the frequency with which they engage in the normal (rather than the clinical) range of alcohol use and other problem behaviors (e.g., delinquent activity). Therefore, the expression of alcohol use disorders among COA’s is considered to be probabilistic, because it occurs at some certainty level less than 100 percent (e.g., Zucker et al. 1995), and not deterministic (i.e., inevitable). Several important scientific questions must be addressed more fully, however, regarding the pervasiveness of alcohol use disorders among COA’s; the identification of major genetic and environmental causes that probabilistically may contribute to the occurrence of these disorders; and the development, application, and evaluation of preventive interventions to ameliorate the frequency and severity of alcohol use disorders and their devastating consequences.

This issue of *Alcohol Health & Research World* focuses on current knowledge about COA’s. In recent years, the number of scientific COA studies has increased dramatically (e.g., Galanter 1991; Sher 1991; Windle and Searles 1990), paving the way for keener insight into the genetic mechanisms and psychosocial processes that contribute to alcohol use disorders among the COA population. Nevertheless, constructive debates among scientists and practitioners coexist with this expanding knowledge base in the ongoing manner that often characterizes the dynamic practice of science. At issue are the relative importance of different etiologic factors (e.g., genetic and environmental), the benefits of alternative COA intervention and treatment strategies, and the advantages of various research and sampling designs. This article first discusses the relative merits and limitations of several study designs in the context of epidemiological issues, then presents a conceptual model that provides a broad perspective on COA functioning. The article concludes with a brief overview of the other articles in this issue, which discuss various factors identified in the conceptual model.

## Epidemiological Issues

Two fundamental, related questions of concern among investigators in the COA field are: (1) how many COA’s are there? and (2) how many COA’s will develop alcohol problems? (Although the frequency of psychological disorders and expressions of maladjustment among COA’s also is of concern, these topics are beyond the scope of this article, which focuses primarily on research investigating the development of alcohol use disorders among COA’s.) Attempts to answer the two aforementioned key questions have resulted in widely varying estimates. With regard to COAs’ risk of developing an alcohol use disorder, estimates have ranged from 4:1 to 9:1 (i.e., COA’s are four to nine times more likely than non-COA’s to develop an alcohol use disorder) (Cloninger et al. 1981; Cotton 1979; Russell 1990), although some researchers have criticized the studies on which these estimates are based (e.g., Murray et al. 1983; Littrell 1988; Searles 1988). Part of the variation in risk estimates is attributable to differences in the criteria used to assess alcohol use disorders in both parents and their offspring. Nonetheless, whether the “true” risk is four or nine times greater (or somewhere in between or slightly less), COA’s unquestionably constitute an at-risk population. Depending on which risk ratio estimate is adopted, however, conclusions may vary greatly about the extensiveness of the health risk among COA’s and the appropriate public health response.

To determine the number of COA’s in the United States, estimates often are extrapolated based on the prevalence of alcohol use disorders among adults in national surveys. For example, Booz, Allen & Hamilton, Inc. (1974) estimated the number of adult alcoholics, then used the ratio of adults to children in the general population to estimate the number of COA’s. Russell (1990) used a similar extrapolation procedure with data collected in the 1979 National Drinking Practices Survey and arrived at an estimate of approximately 6.6 million COA’s under age 18 and 22 million COA’s age 18 and older. More recently, Eigen and Rowden (1995), using data from the 1988 National Health Interview Survey, concluded that 17.5 million COA’s under age 18 lived in the United States. The divergent estimates obtained in these national studies, as well as other estimates obtained through regional or State samples, reflect a range of potential differences in sample selection, data collection strategies, assessment methods, and even definitions of problem drinking or alcohol use disorders. Several major methodological issues contributing to such differences are discussed in the following sections.

### Sampling Variation

Survey sampling involves methods for selecting and observing a part (i.e., sample) of a population in order to make statistical inferences about the whole population. Depending on a study sample’s composition, the prevalence of alcohol use disorders among COA’s may differ across studies. For example, some studies provide estimates based on data collected from patients in treatment for alcoholism. In these studies, the prevalence of alcoholism among parents and other family members is based on the rate reported by the patients in treatment about their families. This sampling procedure likely produces higher than expected (i.e., upwardly biased) estimates of the number of COA’s, because of differences between people in treatment for alcoholism and people in the general population. For example, people in alcoholism treatment are more likely than those in the general population to be disproportionately male, manifest a more severe pattern of alcoholism, have a higher number of co-occurring (i.e., comorbid) psychiatric and medical health conditions, and share other characteristics (e.g., a propensity toward seeking help or involvement with the legal system) (e.g., Heller et al. 1982; Helzer and Pryzbeck 1988). Altogether, such dissimilarities will result in an inflated estimate of the prevalence of COA’s when this sampling procedure is used.

Other studies have estimated the prevalence of COA’s by relying on data derived from samples that were not selected via probability sampling methods^2^ (e.g., volunteers from self-help groups or college students). For example, the use of college students as a sample may underestimate the number of COA’s in the general population, because selection criteria for entry into college (e.g., high academic performance) may disproportionately exclude COA’s.

Probability sampling at the State or regional level is useful for estimating the prevalence of COA’s for a specified sampling unit (e.g., Erie County, New York), but these estimates may not generalize to larger units (e.g., the Nation) because of factors unique to the sampling unit (e.g., Russell 1990). For instance, assume that extrapolation procedures from data on parents in New York are used to estimate the number of COA’s. If the prevalence of alcohol use disorders among parents is greater in New York than in the rest of the Northeast region, then estimates of the number of COA’s based on the New York data would overestimate the prevalence of COA’s in the Northeast or any other less intensive drinking region. Although probability sampling on a national level would eliminate these sampling difficulties, estimates of the number of COA’s and the prevalence of alcohol use disorders among them may still differ across studies because of differences in data collection strategies and the instruments used to assess alcohol use disorders.

### Data Collection Strategies

The method of assessment used to identify alcoholics is another factor that potentially influences the differences across studies in estimates of the prevalence of COA’s. At the family assessment level, two of the methods most frequently employed are the *family study method* and the *family history method*. The family study method involves collecting data (typically through interviews) from *multiple* (or all) members of a family regarding the presence of an alcohol use disorder. Hence, all family members ages 18 and older respond directly to questions about the presence of an alcohol use disorder within themselves as well as within each other family member. In contrast, the family history method involves data collection from a *single* family member regarding the presence of an alcohol use disorder within each family member.

Findings from analyses of studies that used the family history method have been relatively consistent in indicating that this approach underestimates the extent of alcohol use disorders among family members (e.g., Andreasen et al. 1986; Thompson et al. 1982). When rates of agreement on which family members have an alcohol use disorder are cross-checked between target participants (i.e., those who indicate they have an alcohol use disorder) and other family members, the results often range from only 30-to 60-percent agreement (Russell 1990). Furthermore, in cases in which the target participant and other family members disagree, the direction of disagreement is consistent in that other family members tend to indicate lower rates of disorder than the target participants. Thus, family members identified by other family members as having an alcohol use disorder are indeed highly likely to have the disorder. However, a significant percentage of family members with an alcohol use disorder are not appropriately identified with the family history method, which contributes to underestimates of alcoholism in the general population and consequently, the number of COA’s.

Because of the limitations of the family history method, the family study method is a more effective approach for obtaining a reliable assessment of the prevalence of alcohol use disorders. Although this method is preferred scientifically, it is often difficult to implement in practice, because it involves a direct interview with each family member. In addition to the costliness of interviewing each family member directly, difficulties may arise: Family members may be geographically dispersed throughout the country (or world), unwilling to participate, or unable to participate (e.g., because of injury or death). Nevertheless, with sufficient resources, researchers can use somewhat of a hybrid approach between the family study and family history methods to assess the prevalence of alcohol use disorders with reasonable accuracy. For instance, the Collaborative Study on the Genetics of Alcoholism (COGA), a large-scale project initiated by the National Institute on Alcohol Abuse and Alcoholism in 1989 to identify and analyze genetic factors contributing to the risk for alcoholism, attempted to recruit all first-, second-, and third-degree relatives^3^ into the study. However, to minimize the impact of geographical distance as a barrier to participation, the project employed carefully designed selection criteria (e.g., two first-degree relatives of the target participant had to live within 100 miles of a testing center) (e.g., Bucholz et al. 1996).

The use of such a recruitment procedure will likely increase the participation rate by family members and, hence, increase the precision of estimates for alcohol use disorders. Furthermore, precision is improved not only because more family members directly report on their own alcohol use behaviors, but also because the probability of missing a nonparticipating member’s disorder may be reduced if multiple family members report on the nonparticipants’ alcohol use behaviors. For instance, if four family members report on the alcohol use of a fifth member who is not participating in the study, at least two or three of the four respondents may be able and willing to provide accurate data on the nonparticipating member.

To date, most research studies on COA’s have relied on the family history method because of practical constraints. Subsequent research studies, however, may turn to alternative hybrid approaches more frequently.

### Assessment Instruments

Investigators have used several alternative instruments to assess family history of alcoholism, and variability in the scope and precision of these instruments has contributed to different prevalence estimates. The Family History-Research Diagnostic Criteria (FH–RDC) (Endicott et al. 1978), for example, is an interview-based procedure that enables the assessment of alcohol use, other drug use, and psychiatric disorders among family members. In general, research findings support the reliability and validity^4^ of the FH–RDC (e.g., Endicott et al. 1978; Sher 1991). The COGA project, referred to previously, has developed another extensive family history diagnostic interview, the Family History Assessment Modules (FHAM), to facilitate the assessment of psychoactive substance use and psychiatric disorders using the most recent clinical diagnostic criteria (Bucholz et al. 1996). Initial findings with the FHAM have supported its reliability (e.g., Bucholz et al. 1996), and validity studies are in progress.

Screening instruments such as the Michigan Alcoholism Screening Test (MAST) (Selzer 1971) or Short MAST (S–MAST) have been used with young adults to estimate or screen for parental alcoholism (e.g., Sher et al. 1991). High reliability (i.e., interrater agreement^5^) across siblings has been reported for the S–MAST (e.g., Sher and Descutner 1986), and moderate levels of validity have been reported when offspring ratings were compared with parental ratings (e.g., Levenson et al. 1987).

Large national survey studies frequently have used global, single-item questions (e.g., “Has your father ever had a drinking problem?”) to assess family history of alcoholism (e.g., Midanik 1983; Windle 1996*b*). Such single-item assessments of paternal alcoholism have been determined to have reasonably high sibling interrater agreement^6^ (e.g., Sher and Descutner 1986; Windle 1996*b*), but levels of interrater agreement decrease considerably for family members other than fathers (Windle 1996*b*). Thus, using single-item assessments is not recommended if precise estimates of familial alcoholism are desired (Sher 1991).

### Other Contributing Factors

In addition to variations in data collection strategies and assessment methods, several other factors may also contribute to differential estimates of the relative risk for COA’s to develop an alcohol use disorder. To the extent that one or more of these factors is overrepresented in a given sample, estimates of the prevalence of alcohol use disorders among COA’s may be biased. Factors that may increase the risk for alcohol use disorders and thereby contribute to biased estimates include the following:

- *Assortative mating*, which is the nonrandom choice of a partner based on personal characteristics (in this context, alcoholism). For example, compared with female nonalcoholics, female alcoholics partnering with male alcoholics at a greater-than-expected rate increase their offspring’s exposure to risk factors from sources both environmental (e.g., role modeling) and genetic (e.g., Hall et al. 1983). Therefore, prevalence estimates of alcohol use disorders among COA’s would be biased, because the risk for an alcohol use disorder among the subsample with two alcoholic parents is greater than that expected in the general population.
- *Psychopathology* (e.g., clinical depression) in the nonalcoholic partner of an alcoholic parent. Because such a condition may contribute to a disruptive, nonsupportive family environment, its presence may increase risk for alcohol use disorders and other psychopathologies among COA’s (e.g., Windle 1996*a*).
- *Co-occurring psychopathology among alcoholic parents* (e.g., alcoholism coexisting with antisocial personality disorder) (Helzer and Pryzbeck 1988). In these families, heightened genetic and environmental risk (e.g., physical or sexual abuse) may increase the risk for alcohol use disorders and other forms of maladjustment among COA’s.
- *Age, gender, and racial distribution* of given samples. The prevalence of alcohol use disorders is not constant across these demographic variables (Russell 1990). Thus, if the prevalence of alcohol use disorders among COA’s was derived on the basis of a sample of young females, for example, the resulting estimates would be biased if statistical inferences were sought for the general population.

### Summary of Epidemiological Issues

Responses to the dual questions of the number of COA’s and the number of COA’s who will develop alcohol problems may vary considerably depending on multiple factors, including those identified in this article. Several strategies may enhance the consistency of findings across studies, however, such as the use of probability sampling, more precise measurement strategies (e.g., hybrid approaches to the family study method), and measures with demonstrated reliability and validity. Although studies are not in agreement about the estimated magnitude of risk for an alcohol use disorder among COA’s (cf. Murray et al. 1983; Searles 1988), a general consensus does exist that COA status enhances the risk for the expression of alcohol problems to some degree. This higher risk for adverse outcomes among COA’s (e.g., school or work difficulties, involvement with the legal system, and troubled relationships) has spawned a rapidly expanding body of research literature (Sher 1991; Windle and Searles 1990). Through this cumulative research effort, investigators have discerned numerous factors that may influence alcohol-related behavior in COA’s. The remainder of this article discusses a model that encompasses many of these research-identified factors in order to provide an overall picture of COA functioning.

## Conceptual Model of Influences on COA Functioning

About 40 or 50 years ago, theoretical models of alcoholism tended to view the underlying cause of this disorder as most likely influenced by a single factor. For example, causal simplifications, such as “the alcoholic personality” or “the alcoholic gene,” often were promulgated as sufficient explanatory mechanisms for the occurrence of alcoholism. With the proliferation of research studies in the alcohol field in general, and among COA’s in particular, it is now widely accepted that multiple factors influence the onset of alcohol-related behaviors, their progression, and their ongoing status (e.g., continuation of heavy drinking, cessation, and relapse cycles) (Begleiter and Kissin 1995; Sher 1991; Windle and Searles 1990; Zucker et al. 1995). A conceptual model that attempts to incorporate the majority of factors identified as potential influences for adverse outcomes among COA’s illustrates the relationship of these factors (see figure, p. 190).

**Figure:**
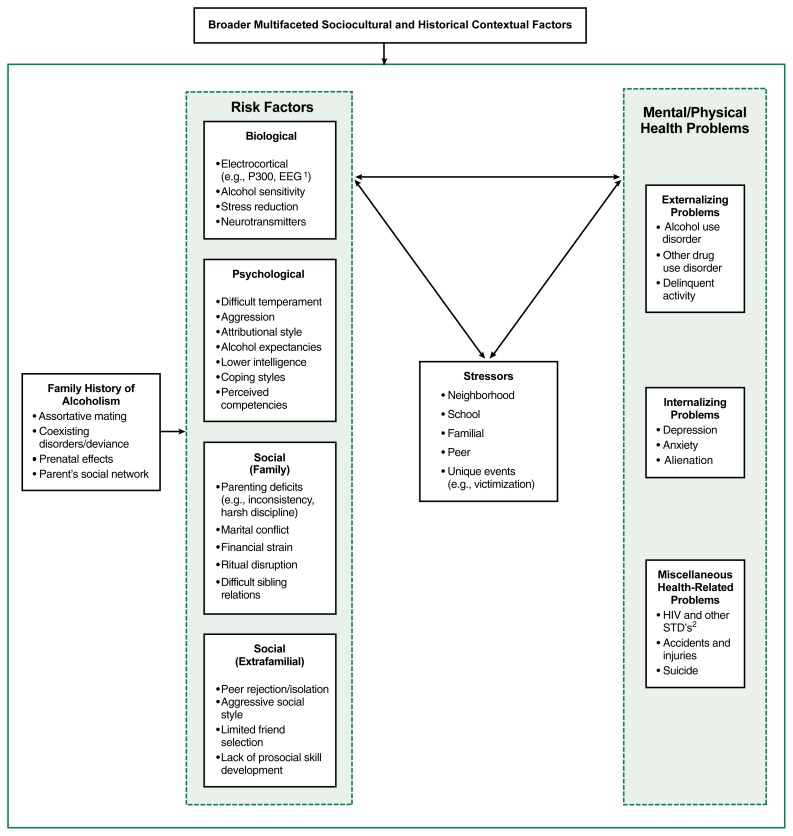
A Dynamic Diathesis-Stress Model of developmental psychopathology as applied to children of alcoholics. This conceptual model offers an overview of how factors related to a family history of alcoholism influence a wide range of other risk factors and may (or may not) lead to the development of psychological or other health problems, including alcohol use disorders. The bidirectional arrows indicate that influences are not one way—that is, risk factors and problems may interact with stressful circumstances and change over time as people influence events around them and as events influence people’s behavior, all within a broad sociocultural and historical context. ^1^EEG = electroencephalogram. ^2^STD’s = sexually transmitted diseases.

Three features distinguish this model, known as the Dynamic Diathesis-Stress Model. First, it is consistent with the general model adopted in psychiatric research of the interaction between a person’s constitutional predisposition to acquire a certain disorder (i.e., diathesis) and outside stressors. That is, this diathesis-stress model recognizes the importance of studying relationships between a person’s characteristics, such as temperament or alcohol sensitivity, in conjunction with stressful environmental encounters that may precipitate the occurrence of a psychiatric or substance abuse episode or disorder. The Dynamic Diathesis-Stress Model suggests that COA’s vary in their relative vulnerability to psychiatric or substance abuse disorders, depending on their individual characteristics, and that the particular life stressors they encounter may trigger the expression of disordered behavior.

Second, the model may be viewed as a multiple-variable (i.e., multivariate) diathesis-stress model, because it recognizes that many personal and environmental factors contribute to ongoing behavioral interactions and given outcomes (i.e., people influence environmental events and environmental events influence people’s behaviors). Focusing on a single risk domain (e.g., parenting deficits) will not likely yield high predictive power for a given outcome (e.g., heavy alcohol use by offspring) or provide a holistic sense of the number of factors (and their interactions) that can contribute to a given outcome. This model, in contrast, accounts for the influence of numerous risk factors.

Third, the model is referred to as “dynamic,” because it explicitly recognizes that the interrelationships between personal and environmental variables change over time and develop into meaningful regularities or cyclical patterns. Whereas the general diathesis-stress model of psychiatric research has focused on a single constitutional factor and a single stress factor at one point in time, the Dynamic Diathesis-Stress Model expresses the interaction of multiple constitutional and stress factors that may reciprocally influence each other across time to produce (or not produce) a given outcome (e.g., an alcohol use disorder). This dynamic orientation has implications for statistical modeling, research design, and substantive interpretation of findings. For instance, prospective research designs, which measure individual subjects repeatedly over time, are essential to capturing the dynamic aspect of the model, because such designs allow a focus on how people change depending on the interaction between their pattern of vulnerabilities and the environmental stressors that occur in their lives.

### A Closer Look

Although this article discusses the Dynamic Diathesis-Stress Model in general terms,^7^ many of the contributors to this issue of *Alcohol Health & Research World* elaborate on findings associated with specific influences identified by the model. Specifically, the article contributions by Larkby and Day and by Jacobson focus on prenatal alcohol exposure and how its effects can be distinguished from effects due to postnatal exposure. As indicated in the figure, prenatal alcohol exposure, like other possible risk factors associated with a family history of alcoholism, may influence functioning in biological, psychological, and social spheres. An article by Jacob and Johnson reviews parenting influences on COA’s and indicates the multiple ways that parental alcoholism may adversely affect child and adolescent development. McGue reviews the expanding literature on the behavioral genetics of alcoholism, which strongly supports a heritable basis for the disorder, and Ellis and colleagues provide a range of family influences that may direct the development of COA’s. Contributions by Finn and Justus, Nixon and Tivis, and Porjesz and Begleiter focus on different response systems (physiological, neuropsychological, and neurophysiological, respectively) that are associated with distinctive at-risk patterns obtained from data on COA’s.

Price and Emshoff describe early intervention and treatment programs designed to prevent substance abuse among COA’s and provide information on screening measures and special treatment concerns. Sher reviews research literature comparing personality, temperament, and childhood disorder differences among COA’s versus non-COA’s, and Reich emphasizes the need for prospective studies that track and periodically interview both COA’s and non-COA’s and their families until the children are past the age at which they are most likely to develop alcoholism. Finally, members of an expert panel provide their views on the “state of the field” in COA studies, placing it in historical context and speculating on promising research directions for the future.

## Footnotes

1 In this article, the term “alcohol use disorder” refers to the entire range of alcohol problems, including alcohol abuse and alcohol dependence as defined by the American Psychiatric Association’s *Diagnostic and Statistical Manual of Mental Disorders*, *Fourth Edition*. The terms “alcoholism” and “alcoholic” as used in this article are summary terms for the diagnoses of alcohol abuse and alcohol dependence.

2 With probability sampling, every member of the population has a known likelihood of being selected as part of the sample.

3 First-degree relatives are mothers, fathers, and siblings; second-degree relatives are grandparents, aunts, and uncles; and third-degree relatives typically are defined as cousins.

4 The term “reliability” refers to consistency of measurement (i.e., the extent to which the measurement procedure yields the same results on repeated assessments). The term “validity” refers to whether a test (e.g., questionnaire) actually measures what it purports to measure (e.g., whether survey items on alcohol use provide good measures of the actual use of alcohol or whether sources of error, such as misrepresentation by respondents, undermine their validity).

5 Interrater agreement is a form of reliability and refers to the degree of agreement or consistency between two or more raters or judges. In this context, interrater agreement provides an index of the level of agreement among siblings regarding their parents’ alcohol use. A value known as a kappa statistic usually is derived to indicate chance-corrected levels of agreement. Kappa values from 0.40 to 0.59 indicate moderate agreement; 0.60 to 0.79, substantial agreement; and 0.80 to 1.00, very high agreement.

6 Kappa statistic = 0.72, indicating substantial agreement.

7 For more information on the Dynamic Diathesis-Stress Model, see Windle and Tubman (in press).
